# Robotic 3D Printing of Geopolymer Foam for Lightweight and Insulating Building Elements

**DOI:** 10.1089/3dp.2023.0183

**Published:** 2024-02-15

**Authors:** Patrick Bedarf, Anna Szabo, Michele Zanini, Benjamin Dillenburger

**Affiliations:** ^1^ Department of Architecture, Digital Building Technologies, ETH Zurich, Zurich, Switzerland.; ^2^ FenX AG, Turgi, Switzerland.

**Keywords:** construction automation, foam 3D printing, sustainable materials, responsible consumption

## Abstract

Foam 3D printing in construction is a promising manufacturing approach that aims to reduce the amount of material, hazardous labor, and costs in producing lightweight and insulating building parts that can reduce the operational energy in buildings. Research using cement-free mineral foams derived from industrial waste showed great potential in previous studies that reduced the amount of concrete needed in composite structures. This article collates the latest developments in this line of work. It presents the material system with its principal components and the advanced robotic 3D printing setup with a climate-controlled fabrication chamber. Print path schemes and hybrid fabrication methods combining 3D printing and casting are evaluated. Furthermore, the article discusses the effect of different print path schemes on the thermal insulation and compressive strength performance of printed parts. A full-scale final prototype synthesizes these findings and demonstrates the fabrication of modular, lightweight, and insulating construction elements that can be assembled into monolithic wall structures. The advantages and challenges of this novel approach are elaborated on in the conclusions. Finally, the article presents future advancements required to leverage this research as a scalable construction method that can help address the biggest challenges in building low-carbon and energy-efficient structures.

**Figure:**
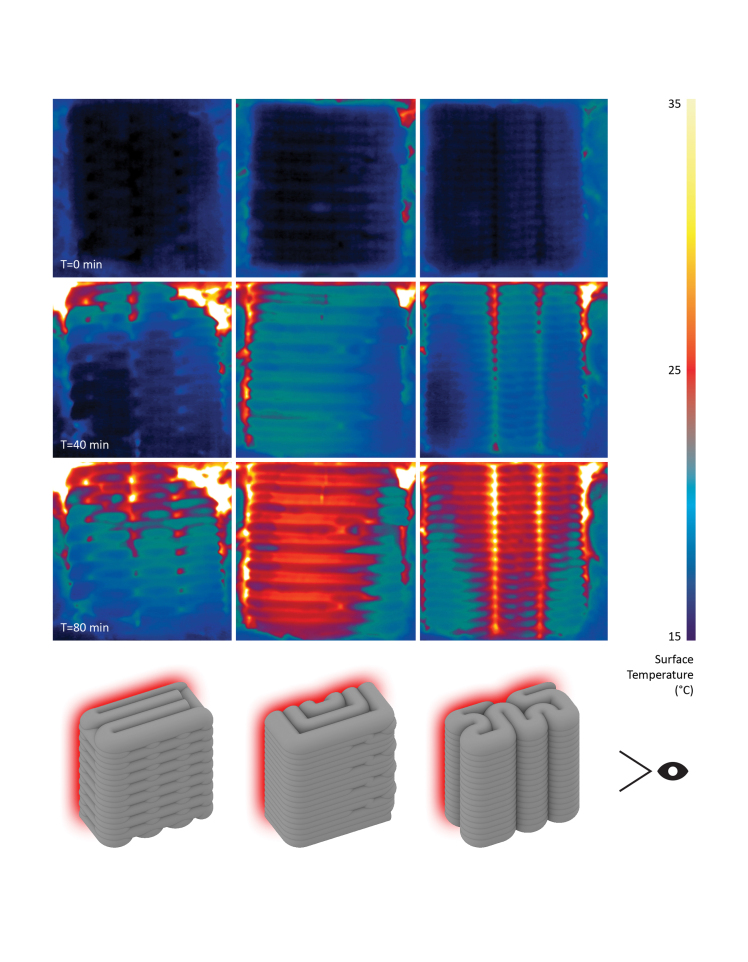


## Introduction

Construction 3D printing has evolved into a comprehensive research field during the last two decades and has started impacting the building industry. What began as an appropriation of rapid prototyping for disaster relief is today a thriving community of academic and commercial actors who advance the technology and materials, achieving new records of printed structures at an accelerating pace.^1,2^ The primary motivation behind these efforts relies on the potential offered by construction 3D printing and robotic fabrication to help mitigate the biggest challenges in the building industry and reduce material use, embodied carbon emissions, hazardous labor, and costs.^3,4^

Concrete 3D printing is a disruptive technology in this context because of the high compressive strength of the resulting prints and the potential to save resources by fabricating geometrically optimized, material-efficient structures that were previously cost-prohibitive with conventional building techniques.^5^ Although these are urgent and laudable developments, concrete 3D printing has still not expressed its full potential. In fact, printed structures commonly feature simple geometric shapes that are not optimized for material efficiency, print materials often have a higher carbon footprint than regular concrete, and printing equipment is not readily available or only at a high initial cost.^6^

In contrast to construction applications requiring pure strength, foam 3D printing (F3DP) enables the automated and moldless fabrication of lightweight and insulating building elements.^7^ Using the properties of porous printing materials allows to create novel building components that require fewer materials to make, have a lower embedded carbon footprint and mass, and help to decrease the operational energy for heating and cooling. Therefore, F3DP can contribute to significantly reduce the emissions of both material and energy consumption in the building industry.

### State-of-the-art

Automated construction techniques involving foamed materials have been documented since the early 1960s. The porous insulating materials used in the field of F3DP can be categorized into two distinct types: organic and inorganic foams. The first is commonly recognized as plastic foams, such as polystyrene (PS) or polyurethane (PU), and the second encompasses various cementitious, clay-based, and geopolymer foams. Regarding environmental sustainability, inorganic foams are regarded as the superior choice due to their reliance on nonpetroleum-based raw materials. In addition, they conventionally do not present any toxic emissions and are inherently nonflammable.

Several recent projects explored F3DP in construction with plastic foams. Researchers developed an autonomous robotic fabrication platform, including a PU spray printing system, and demonstrated the fabrication of an architectural-scale hemi-ellipsoidal dome section spanning 14.6 m in diameter.^8^ In Nantes, researchers of the BatiPrint project printed the stay-in-place molds for concrete casting of a 95 m^2^ building using automatic spray printing of PU foam.^9^ Plastic foams are often used as filler in printed structures such as hollow walls and spatial lattices to increase the thermal resistance of the finished building component. Alternatively, bio-based, renewable alternatives are investigated in research on large-scale F3DP of composite structures and are envisioned to solve the sourcing problem of organic foams.^10^

Complementary to organic foams, inorganic counterparts have also found their way into large-scale F3DP research. Cement foams are the most commonly investigated material, and their formulations, properties, and applications are extensively explored.^11^ Researchers used lightweight cement-based foams to fabricate wall elements with cellular cross-sections to optimize their insulation properties.^12^ Functional grading with different densities of cement foams was demonstrated for robotic F3DP with the potential application for ribbed slab and cavity wall elements.^13^

However, the cement content significantly increases the embedded carbon footprint of these structures, and alternatives could be researched. For example, clay-based foams sintered for consolidation were used with F3DP in another study to produce hierarchical porous ceramic bricks with programmed thermal insulation and evaporative cooling effects.^14^ Sintered mineral foams were utilized with F3DP to show their potential combined with concrete for facade and beam structures.^15^ The energy-intensive sintering step could be omitted using set-on-demand geopolymer-hardened mineral foam to 3D print the functional stay-in-place formwork of a ribbed concrete slab prototype.^16^

### Contribution

This article highlights the latest research in F3DP with cement-free geopolymer-hardened mineral foams based on industrial waste for the automated, moldless fabrication of modular, lightweight, and insulating wall elements. The material system, codeveloped with the sustainable insulation specialists of FenX AG, is presented. An overview and in-depth description of the robotic F3DP setup are provided, including the advancements of a climate-controlled fabrication environment and geometric print path planning. The discussion includes the evolution of prototypes and their structural and thermal evaluation. Finally, the final demonstrator of a 2 m tall wall assembly of four 3D-printed segments is presented. The conclusions reflect upon the achieved milestones in this research and indicate required improvements for future work. This article discusses the significant contributions that F3DP can make toward addressing the environmental and economic challenges in the building industry. By highlighting its potential, this research underscores the importance of F3DP in promoting responsible material and energy usage in buildings, offering a promising outlook for sustainability efforts in construction.

## Methods and Materials

### Material system

The fully continuous F3DP process presented in this article is performed with a cement-free set-on-demand geopolymer foam controlled in its hardening. The main components of this foam are fly ash, water, and air, complemented with retarders, mineral additives, and a hardener. The retarders are responsible for the long open time of the slurry before printing, and the combination of the mineral additives and the hardener controls the foam transitions from a yield stress fluid to a cohesive solid material.

The basic requirements for the slurry were fulfilled by adjusting the mix design in three steps. First, calorimetry was used to study the open time needed for large-scale fabrication of the base slurry (slurry without air and hardener). Second, slump tests were conducted to assess the changes in the material over time when the hardener was added to the base slurry. Based on the learnings from calorimetry and slump tests, the robotic 3D printer has been set up. The dosages have been adjusted based on empirical evaluation of printability and buildability. The developments of the material composition reported in this article are shared intellectual properties between ETH Zurich and FenX AG. Thus, their description will be kept conceptual.

**FIG. 1.**
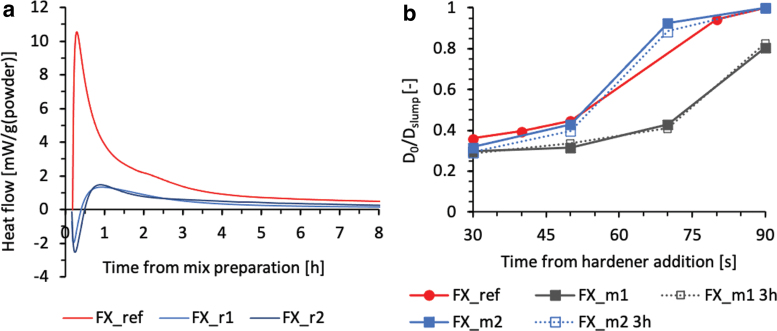
**(a)** The effect of retarder addition measured with calorimetry. The FX_ref is the reference slurry without retarders. FX_r1 and FX_r2 mixes contain different amounts of retarder. **(b)** Slump tests over time of hardening slurries (Representation with *lines* as visual guides).

Isothermal calorimetry was conducted with an I-Cal8000 at 23°C to study the qualitative reaction kinetics of the base slurries without hardener by their released heat over time. Figure 1a shows that the reaction rate could be slowed significantly for the first 3 h when the retarder dosage is in the range defined by FX_r1 and FX_r2. Consequently, the base slurry can be prepared as a big batch and used gradually for a day-long F3DP experiment.

Slump tests with cylinders of *d* = 50 mm and *h* = 50 mm dimensions were used to investigate the rate of strength evolution of hardening slurries. Four cylinders were filled immediately after mixing the hardener with the base slurry, and they were lifted after 30, 50, 70, and 90 s of rest. The FX_ref slurry is the reference. All samples tested have the same hardener dosage. FX_m1 and FX_m2 base slurries contain different amounts of mineral additives. To validate the capability of the base slurry to be activated from a dormant state on demand, two sets of slump tests have been carried out. In the first run, FX_m1 and FX_m2 were activated immediately after homogenization. In the second run, the same compositions were the base slurry that got activated after 3 h of rest. Figure 1b depicts the slump results as the relative spread of slurries as a function of the contact time of the base slurry with the hardener. The material retains full shape for values of Dslump/D0 equal to 1. Interestingly, FX_m1 and FX_m2 have similar hardening behavior during their open time. FX_m1 is considered too slow comparatively, while FX_m2 presents an adequate timescale for shape retention in an industrial F3DP process.

### Robotic 3D printer

**FIG. 2.**
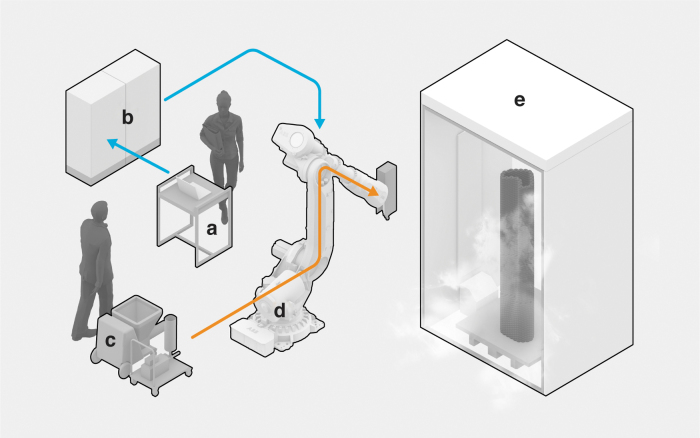
The robotic F3DP setup: **(a)** robot and print head operator, **(b)** robot and printhead controller, **(c)** slurry and hardener feed, **(d)** industrial robot with the printhead, and **(e)** climate-controlled chamber (flow of data shown in *blue*, material in *orange*). F3DP, foam 3D printing.

The robotic F3DP setup is used to continuously mix a stream of base slurry with hardener and air, extrude it to a foam beam, and print it (Fig. 2). The setup consists of an ABB IRB6700 high-performance six-axis robotic arm with a 150 kg maximum payload and 3.2 m of maximum reach (Fig. 2d). The end effector of the robot is a printhead customized for geopolymer F3DP with 20 kg of weight. The printhead acts as a mixing and foaming chamber that can be operated at a max of 5500 rpm. High rpm, thus high shear, is advantageous for foaming. However, this feature makes liquid cooling as temperature regulation indispensable to maintain quasi-constant experimental conditions. To provide flexibility, the printhead has individual inlets secured by check valves to receive the continuous flow of foam constituents separately.

The flow of the base slurry, the hardener, and the air is regulated by the peripheral equipment (Fig. 2c). A PFT SWING M progressive cavity pump is responsible for forwarding the base slurry, an eccentric worm pump is dosing the hardener, and the pressurized air is regulated by an airflow control valve. The individual control over the flow of these components defines the extrusion speed of the foam filament and provides freedom to achieve faster or slower hardening or different foam densities. Typically, 400–500 kg/m^3^ densities were achieved with 21.34 Hz motor frequency for slurry, 40 Hz for hardener, and 1.5 ln/min air. These control parameters are sent through a custom PLC controller (Fig. 2b), while the print path is sent from a Rhino+Grasshopper environment to the robot controller using the COMPAS framework (Fig. 2a).

The typical fabrication sequence of a print experiment is the following: first, a large batch of retarded base slurry is prepared with a planetary mixer. Then, the slurry and hardener are fed into the F3DP setup. The print head is operated stationary, and parameters are adjusted until a satisfying filament is achieved. The printability of the material, combining foam stability, extrudability, and shape retention, is assessed visually. Afterward, the robotic manipulator transports the print head along all print path locations until the print part is completed. Buildability is evaluated with prototypes consisting of multiple layers, where the lack of failure mechanisms, such as deformations, buckling, or cracking, indicates success. After fabrication, the finished part stays in the climate-controlled chamber for 1 week with programmed temperature and relative humidity (Fig. 2e). Finally, the part stays another week at ambient room temperature and humidity.

### Curing environment

A strategy had to be devised to address early volumetric change in the deposited material. This is especially important for 3DP with foams because the entrapped gas is subjected to a significantly larger temperature-induced volume change than solids. To minimize the associated strains and to control the drying rate, the printing had to be carried out in a fabrication environment where it is possible to adjust temperature and relative humidity. This tempered environment would be beneficial during fabrication and for programming the environmental conditions throughout the hardening by geopolymerization.

The environmentally controlled fabrication chamber in this study consists of a lightweight framed tent structure with the dimensions of 2.8 × 2.0 × 1.5 m that is clad with vapor barriers and thermal insulation boards. One side of the chamber is equipped with a lamella curtain to allow the robotic arm of the printer to access the print area. Two household humidifiers are used to modulate the relative humidity with integrated humidity sensors and negative feedback control. Four infrared heating panels with a total power of 1700 W are mounted to the side walls to adjust the temperature.

**FIG. 3.**
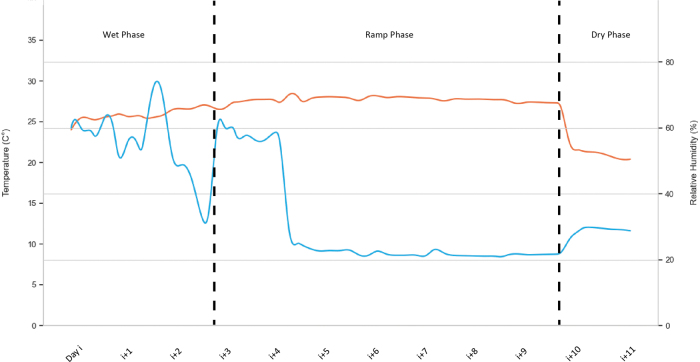
Data log of climate-controlled fabrication environment showing temperature (*red*) and relative humidity (*blue*) changes over a period of 12 days.

The performance of the climate control system was monitored by a handheld data logger sensor placed at the bottom of the print area. The retrieved values of temperature and relative humidity can be seen in Figure 3. Across the entire cycle, the temperature ranged from 20°C to 28°C and relative humidity between 20% and 80%. Fluctuations in humidity control occurred from the humidifiers running out of water and their unreliable feedback control. Consequently, these deviations affected the temperature as well. Figure 3 shows an excerpt of the temperature and relative humidity development during the first week after fabrication with high humidity (wet phase), the second week with decreasing humidity (ramp phase), and the phase at ambient conditions.

### Print path schemes

Several print path designs were developed in this research of F3DP with cement-free mineral foams for large-scale building elements with solid infill.^15^ Direction-continuous spiral, contour-perpendicular zigzag, and alternating direction-parallel zigzag infill schemes were considered as options for the present study. However, to demonstrate the potential of this research for larger modular wall structures, the scale of printed elements had to be increased significantly while maintaining a low mass for lightweight manual assembly. These requirements necessitated a different manufacturing approach that combines thin shell printing with casting to achieve a foamed mono-material system.

**FIG. 4.**
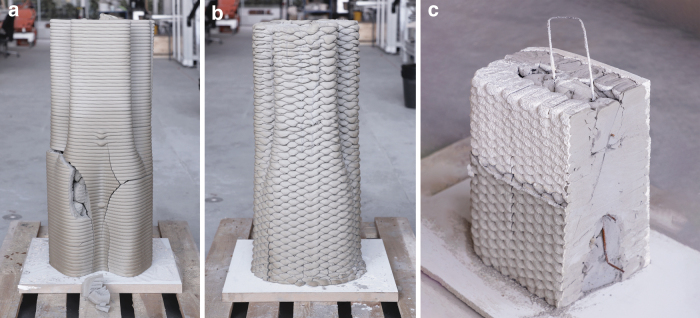
Print path prototypes: **(a)** single-wall contour shell design, **(b)** contour-perpendicular zigzag shell, and **(c)** a cut specimen combining printed shell with cast infill, including rebar and sprayed plaster cover.

During prototyping, a 1 m high single-wall contour shell design was 3D printed, and the inner void was filled with cast mineral foam (Fig. 4a). The specimen showed no visible cracks during fabrication and curing in the wet state. However, when transitioning to the dry state, fine cracks occurred and increased in size during the subsequent days. To reduce the risks of cracks, the shell was thickened and reinforced with a corrugation pattern adapted from the contour-perpendicular zigzag infill scheme. A second 1 m high prototype confirmed the success of this approach in showing no crack formation after completing the curing cycle (Fig. 4b). A third prototype was fabricated that combined the chosen corrugated print path design, including a rebar inlay with a lifting handle detail, cast foam inside the central void, and a protective spray-plaster layer on the exterior surface to increase impact resistance (Fig. 4c). The plaster finish can be tuned in thickness and smoothness depending on the application. It concluded that the chosen printing strategy, casting, and surface coating work harmoniously and can lead to a functional real-scale demonstrator.

## Results and Discussion

### Compressive strength

**FIG. 5.**
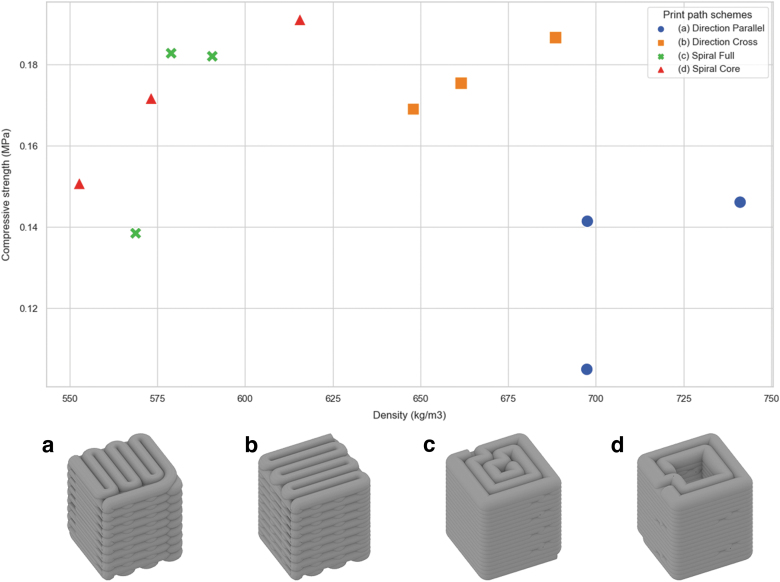
Compressive strength test of F3DP specimen with different infill schemes: **(a)** Direction-parallel, **(b)** direction-parallel cross, **(c)** direction-continuous spiral, and **(d)** double-wall contour-parallel shell with cast inner void.

Preliminary compressive strength tests with the casted mineral foam into 5 × 5 × 5 cm standard cubes resulted in an average capacity of 2 MPa and a density of 500 kg/m^3^. However, spatially arranging the material in an F3DP process induces additional interlayer porosity and anisotropy. Consequently, 3D-printed specimens had to be evaluated. The tested infill schemes are shown in Figure 5 and comprise (a) direction-parallel, (b) direction-parallel cross, (c) direction-continuous spiral infill, and a sample combining a 3D-printed (d) double-wall contour-parallel shell with a cast inner void. The samples with target dimensions of 20 × 20 × 20 cm were produced in two batches: direction-parallel infill in the first batch and all others in the second.

After the curing cycle, the compressive strength testing was conducted on a type Walter & Bai 502 machine with Proteus MT software. Samples (a) failed at peak force between 4.2 and 5.8 kN, (b) between 6.5 and 7.1 kN, (c) between 5.0 and 6.6 kN, and (d) between 5.3 and 6.9 kN. Table 1 shows the sample data, including the dimensions, mass, and measured peak force. The distribution of resulting compressive strength in relation to the sample density can be seen in Figure 5. A fifth series of tests were conducted to validate the experiment.

**Table 1.** Compressive Strength Test of Foam 3D Printing Specimen

Print path scheme	Sample No.	Length (mm)	Width (mm)	Height (mm)	Mass (g)	Density (kg/m^3^)	Force (kN)	Compr. strength (MPa)
(a)	1	200	205	213	6092	698	5.8	0.14
	2	200	200	215	5998	697	4.2	0.11
	3	200	195	210	6070	741	5.7	0.15
(b)	1	195	195	210	5498	689	7.1	0.19
	2	190	195	208	5099	662	6.5	0.18
	3	210	200	214	5824	648	7.1	0.17
(c)	1	190	190	207	4326	579	6.6	0.18
	2	190	190	210	4312	569	5.0	0.14
	3	185	190	205	4256	591	6.4	0.18
(d)	1	190	190	196	4356	616	6.9	0.19
	2	185	190	210	4080	553	5.3	0.15
	3	190	190	205	4242	573	6.2	0.17

Overall, the experimental results show that 3D-printed samples exhibit a significantly lower compressive strength with 0.14–0.21 MPa than cast specimens. Furthermore, no significant differences in performance between the different infill schemes (a–d) can be observed. Samples with direction-parallel infill (a) result conversely weaker. However, their significantly higher density indicates a higher water content that might have resulted from an incomplete curing cycle of the first print batch.

### Thermal insulation

Mineral foams can be excellent thermal insulators. Their performance greatly depends on their density. Thermal conductivity for a cast mineral foam with a density of 70 kg/m^3^ and a composition similar to the one used in this study is lower than 0.04 W/mK. The material used for F3DP features higher densities and, therefore, insulation performance. However, a qualitative thermographic experiment was performed to understand the influence of print path schemes on thermal resistance.

**FIG. 6.**
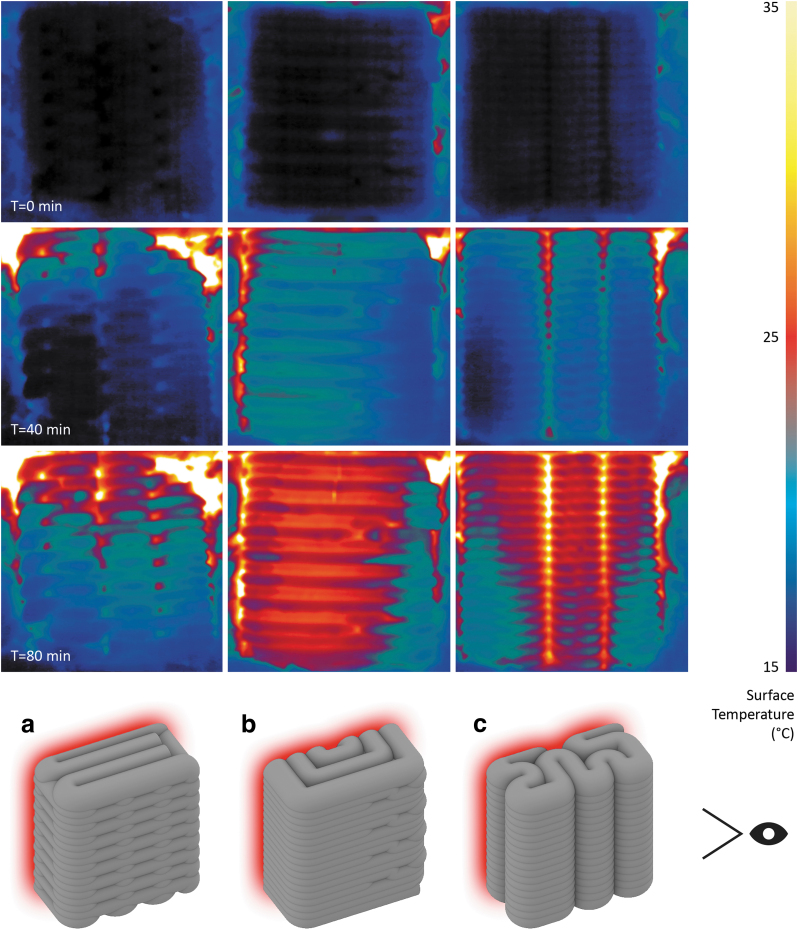
Thermographic comparison of infill schemes after 0 (*first row*), 40 (*second row*), and 80 min (*third row*): **(a)** direction-parallel cross, **(b)** direction-continuous spiral, and **(c)** contour-perpendicular zigzag. Print path schemes are illustrated in the *bottom row* with the heat source indicated in *red* and the camera position by the eye symbol.

Cubic specimens measuring 20 × 20 × 10 cm with the following print path schemes were produced: (a) direction-parallel cross, (b) direction-continuous spiral, and (c) contour-perpendicular zigzag (Fig. 6). Schemes (a) and (b) were chosen because of their high compressive strength results and (c) as a result of prototyping highlighted in the section on print path schemes. The samples were encapsulated with 50 mm mineral wool and 25 mm wood composite board. The back face was exposed to a 400 W silicone heat mat connected to a PID temperature controller set to the target temperature of 300°C. The front face of the samples remained exposed and was recorded for 80 min by a FLIR infrared camera.

### Final prototype

**FIG. 7.**
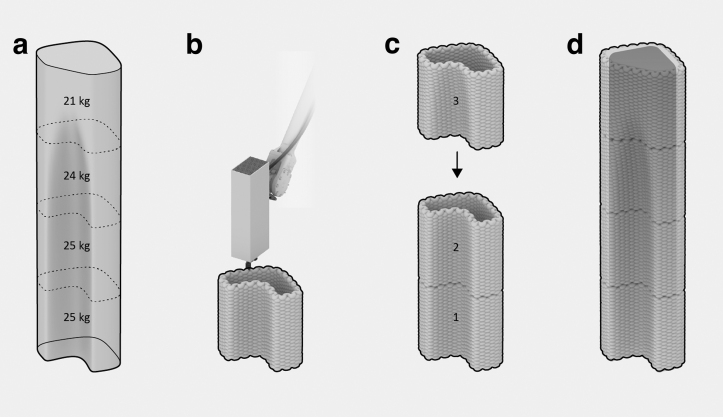
Design and fabrication sequence: **(a)** global geometry segmentation, **(b)** 3D printing of segments, **(c)** manual assembly of segments, and **(d)** casting foam into the central void.

The final prototype examined in this investigation pertains to constructing monolithic and insulating walls featuring a custom-shaped, alcove-like geometry within a 2-m-tall corner configuration. It comprises four hollow elements stacked on top of each other, each measuring 50 cm in height, with an approximate footprint of 40 × 40 cm. These elements possess a weight ranging between 21 and 25 kg, enabling a single individual to assemble them effortlessly. To achieve a unified structure, mineral foam can be poured into the central cavity, resulting in a monolithic formation (Fig. 7). This approach also facilitates the seamless integration of reinforcement and other infrastructural installations. Although a smaller single-segment prototype was utilized to validate both steps, their implementation was omitted for the full-scale assembly. Instead, this study focuses on evaluating the resilience of the F3DP method, completing the assembly utilizing conventional construction equipment, and the attainment of a high-quality surface finish suitable for facades, achieved through the application of cement-free plaster spraying.

**FIG. 8.**
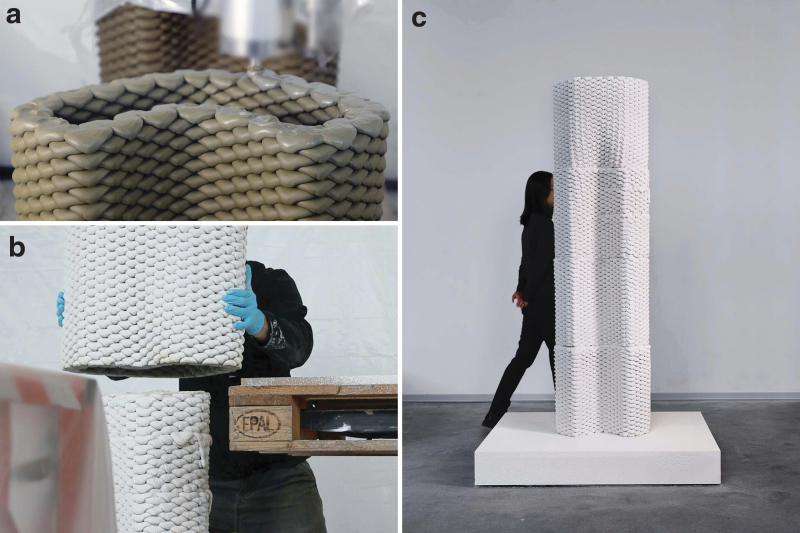
The final prototype showing **(a)** 3D-printing process, **(b)** manual assembly, and **(c)** the finished structure.

After discretizing the overall geometry into four parts, all segments were printed in two consecutive sessions. Each print session lasted ∼1 h for material preparation and 40 min for printing both parts (Fig. 8a). After curing, the segments were stacked vertically using cement-free, lime-based mortar as a thin interface layer (Fig. 8b). One person could complete the assembly using standard, light-duty construction equipment, taking ∼45 min. Finally, a thin layer of cement-free, lime-based plaster was sprayed onto the assembly to enhance impact resistance, a conventional step for using insulation materials in exterior walls. Although foam casting into the hollow core was successfully tested in a previous experiment, this step was not necessary for the successful realization of the demonstrator (Fig. 8c).

## Conclusions

F3DP in construction is an innovative manufacturing method with the potential to make buildings with fewer resources and lower operational energy consumption. This article collates the latest developments in the research using cement-free geopolymer-based mineral foams. The material and large-scale robotic F3DP system are presented. Optimal temperature and relative humidity conditions are provided inside a climate-controlled fabrication environment. A final prototype demonstrates the combination of F3DP and casting of mineral foam assembled into monolithic wall structures. The tested compressive strength of specimens made by F3DP indicates the main areas of applications. Monolithic elements produced by printing and casting mineral foam are suitable for nonstructural wall and facade elements. When printed foam elements are combined with concrete as structural filler, the resulting parts can be used for structural applications such as load-bearing walls and slabs. In both cases, this method allows the automated and waste-free fabrication of lightweight and insulating building elements with custom geometries. The qualitative effect of different infill print path schemes on thermal insulation performance could be observed. The first conclusions could be made on the effect of alternating filament orientations in relation to the thermal gradient.

Future research in this field can focus on automation, process robustness, and developing novel applications that leverage advanced print path schemes to optimize thermal and structural performance with varying foam densities. Process automation may entail the control integration of the robotic manipulator, printhead, and peripheral pumps into a unified interface. This advanced level of control would enable precise tuning of foam densities during the printing of graded material structures. Furthermore, start-stop printing operations would allow more geometric freedom in the design of print path schemes. Together, those advancements increase precision for meeting required construction tolerances.

The climate-controlled fabrication chamber can be improved to better program relative humidity and temperature levels. The tent can be resized according to the size of the print parts while allowing the robotic printer to reach the print area. Multiple chambers could be accessed by a mobile F3DP system to parallelize fabrication and increase productivity. Another approach could integrate the robotic manipulator into the climate-controlled environment altogether as a compact manufacturing solution.

As an outcome of this research, the modular wall assembly prototype reveals future potential for F3DP in construction. If the compressive strength of printed structures can be increased, structural applications for monolithic construction become possible. The prototypes in this article are the first indication of the performance of building components produced solely by F3DP. Understanding the interrelation of print path schemes with structural and thermal performance should be deepened to develop systematic design guidelines for printed parts. This understanding can lead to the next generation of resourceful and energy-efficient structures for future sustainable construction.
